# Agricultural load cycles: Tractor mission profiles from recorded GNSS and CAN bus data

**DOI:** 10.1016/j.dib.2025.111494

**Published:** 2025-03-20

**Authors:** Korbinian Götz, Andrew Kusuma, Adrian Dörfler, Markus Lienkamp

**Affiliations:** TUM School of Engineering and Design, Chair of Automotive Technology, Technical University of Munich, Boltzmannstr. 15, Garching, 85748, Germany

**Keywords:** Real-world data, RTK, GNSS, GPS, Mobility data, Fleet data, Data mining

## Abstract

Agriculture is one of the main sources of greenhouse gas emissions, with agricultural machinery playing an important role through the combustion of fossil fuels. On the upside, many farmers invest in their own renewable energy generation. To leverage the renewable produced energy and cut emissions, a promising solution is the electrification of tractors. With the advent of tractor electrification, the necessity to define the requirements of an electrified tractor emerges. In contrast to automotive and truck engineering, where the usage patterns are narrow, and testing is standardized through load cycles as the WLTP and VECTO LongHaul, the agricultural application of tractors includes a wide variability of operating conditions. Since there are no standardized, publicly available agricultural load cycles, we equipped, during a one-year period, five tractors (77–240 kW) on a farm in the South of Germany with high-resolution GNSS loggers and collected the CAN bus data from the J1939 engine bus and the ISOBUS. The data collected is enriched with information about the tractor models and implements used. Hence, the load and usage information in real-field operation is suitable as input for the development of electrified tractors.

A total of 862 h of operation data has been recorded and constitutes one of the most comprehensive open datasets to date for high-resolution data of agricultural tractors. This dataset provides engine and load parameters, auxiliary power supply, and GNSS information for multiple different agricultural fields. Its applications include the simulation of electrification for agricultural tractors, load cycle construction, modeling of farming processes, the analysis of agricultural production indicators, or path planning for autonomy.

Specifications Table

**Table:** 

Subject	Engineering & Materials science
Specific subject area	Real-driving GNSS and CAN-Bus Recordings, Mobile Machinery Electrification, Agriculture
Type of data	Mobility Data Table Filtered
Data collection	The data was recorded using CAN data loggers with GNSS capability. We installed the loggers 5 different tractors (77–240 kW) on the farms of the Technical University of Munich to record movement and CAN bus data in the 2024 agricultural season. The bus loggers were connected to the Society of Automotive Engineers (SAE) standard J1939 CAN bus and the ISO 11783 standard (ISOBUS). We automatically filtered the data for the fields worked and enriched the data set with information about the tractors and implements used. In order to anonymize the dataset for public availability, the actual time of recording is hidden.
Data source location	Institution: Technical University of Munich, TUM School of Engineering and Design, Chair of Automotive Technology, D-85748 Garching, Field Crops Unit, TUM Plant Technology Center, D-85354 Freising Collection sites:
	48.18005350833466° N, 11.320330776532794°
	E 48.40614225784452° N, 11.702033416499619° E
Data accessibility	Repository name: Zenodo Data identification number: 10.5281/zenodo.14619787 Direct URL to data: https://zenodo.org/records/14619787 Github repository: https://github.com/korbig/TractorCAN-GPS Instructions for accessing these data: The Zenodo repository contains a set of compressed CSV files. The github repository contains a jupyter notebook to get a view into the data.
Related research article	none

## 1 Value of the Data

• To the best of the authors’ knowledge, this is the first publicly available dataset of tractors in agricultural operation, which reflects real-world operation with a multitude of different tractors and implements. Because no representative load cycles for agricultural machinery are standardized or publicly available, researchers lack access to resources to analyze tractor efficiency and productivity. Our open-source dataset provides this opportunity to every researcher and developer while bypassing the time and cost-intensive data collection step.
• The dataset provides operational data for tractors with implements in the 77–240 kW power range to minimize the potential mismatch between the load cycle and real-world application. It provides knowledge on agricultural operations, reflecting different cultivation methods and implement sizes.
• In contrast to data sets with pure GNSS information, this data record contains the high-precision GNSS movement paired with the relevant engine signals for querying load and power as well as bus signals about the status of the implement, such as the PTO shaft, three-point linkage, and draft force. To enable the transfer to real-world loads, the data was enriched with information about the tractor and implement used during the recordings (e.g. power harrow, seed drill combination, plough, fertilizer, mower, and silage transport). The data allows the researcher to identify the operation and load modes of the machines as well as impacts from wheel slip and implement size.
• The dataset excels in GNSS accuracy as real-time kinematic (RTK) correction services with ± 2 cm accuracy were used. The availability of the not corrupted GNSS location allows it to be combined with satellite imagery, while the high resolution enables the retrieval of productivity indicators or field characteristics determining the path planning. This is of particular interest to researchers investigating land use.
• The data is of interest to researchers and professionals in agricultural engineering who are investigating the requirements for tractor powertrains or determining load cycles for the electrification of novel agricultural machinery. The data is suitable for developing automated data mining methods to retrieve machine states, productivity data, or operational cost values.

## 2 Background

The transition to electrified agricultural machinery requires solutions tailored to farmers, leveraging the design freedom of electric drivetrains [1]. Key to this is understanding tractor mission profiles and load demands during various operations [2]. According to Scolaro et al. [3] and Mocera [4], the lack of a reference load cycle is one of the major obstacles to tractor electrification due to the missing knowledge to size electric drives.

In contrast, automotive engineering benefits from standardized test cycles like WLTP for cars and VECTO Long Haul for trucks, supported by publicly available fleet data. These enable the design and optimization of electric powertrains [[5], [6], [7]]. Agricultural engineering lacks the resources of publicly available reference load cycles. Existing cycles, such as the OECD Tractor Code 2 and NRTC^1^, focus solely on diesel engine emissions and fail to account for the diverse operational profiles of tractors. Tractor loads are influenced heavily by the implement and soil conditions, forcing researchers to rely on limited self-recorded data [[8], [9], [10], [11], [12]], which restricts analysis and comparability. Additionally, mission profiles and requirements of tractors are wide, changing by farm type, location, and user. The electrification must take the great variability of load and performance requirements into account to provide suitable concepts for a significantly large group of farmers [13]. Key to an efficient and cost-optimal design of a novel electric powertrain is the knowledge of the various applications and mission profiles for different implements.

Ettl et al. [14] developed test cycles based on 200 h of real-world data, covering six implements and combining drivetrain, PTO, hydraulics, and auxiliary power requirements. Still, the data is limited to one specific tractor size and contains only six different implement sizes. Similarly, the DLG PowerMix test evaluates energy consumption in 14 scenarios [15], but, according to Angelucci and Mattetti [2] omits idling and is inaccessible for research.

This dataset was created to provide a detailed view of tractor loads throughout one season and suits to develop optimized electric tractor concepts. The total amount of working hours and the variety of our dataset exceeds the previously mentioned cycles from Ettl et al. [14], the PowerMix [15], and Angelucci and Mattetti [2].

## 3 Data Description

The folder structure of the data is organized by the recorded tractor, attached implement, and the worked field. Each tractor includes the data in two variants: (1) a dataset including all recordings (*{Tractor model}*.csv) as well as (2) the data sorted by the operated implement and automatically filtered for the worked fields (*{Field_id}.csv).*

Table 1 shows a full summary of the recorded tractors, with the number of worked fields, and the recorded duration of the used implements in the dataset. To obtain the dataset, a total of 862 h and 31034711 data points were recorded. With the automated methodology (EXPERIMENTAL DESIGN, MATERIALS AND METHODS), the published data comprises 354 h of pure fieldwork on 143 fields.

**Table 1:** Information on the logged tractor models associated with their used implements.

Tractor model	Tractor power [kW]	Work type	Number of fields	Recorded hours [h]	Used implements/working width [m]
Fendt 211 Gen3	77	Power harrowing	2	2.1	Lemken Zirkon 3 m
		Precision air seeding	2	0.7	Monosem 3 m

Fendt 314 Gen4	104	Fertilizing	14	7	Rauch Axis 15 m
		Spraying	13	6.2	Gaspardo 15 m
		Seed drill combination	15	38.3	Pöttinger power harrow & seed drill 3 m
		Disc harrow	3	5.5	Horsch Joker 3 TC 3 m
		Mowing (front)	1	0.5	Fendt Slicer 3060 FP 3 m
		Mowing (large-scale)	3	1.7	Claas Disco Trend 8500 8.3 m
		Swathing	2	3.2	Deutz dual-rotor swather SwatMaster 6951 6.9 m
		Silage transport	3	16.7	Lang tipper 13 tons

Fendt 820	140	Cultivating (deep)	8	26.4	Kerner Komet K420 4.2 m
		Mulching	8	3.6	3 m
		Rotary tiller	3	0.9	3 m
		Seed drill combination	1	0.9	Amazone D9 4000 Super 4 m & Lemken Zirkon 4 m
		Seedbed combination	1	2.3	Lemken Korund 6 m
		Ploughing	3	4.7	Lemken Europal 8 with 5 ploughshares 2 m

Fendt 722 Gen6	163	Fertilizing	6	4.9	Rauch 24 m
		Seedbed combination	3	19.3	Lemken Korund 6 m
		Cultivating (deep)	4	18	Kerner Komet K420 4.2 m
		Cultivating (shallow)	5	15.6	4 m
		Power harrowing	3	4.5	Lemken Zirkon 4 m
		Disc harrowing	3	6.7	4 m
		Mulching	7	23.9	3 m
		Ploughing	6	40.5	Lemken Europal 8 with 5 ploughshares 2 m
		Seed drill combination	6	38.9	Amazone AD 303 3 m & Amazone KG 302 3 m
		Seed drill combination	6	19.4	Amazone D9 4000 Super 4 m & Lemken Zirkon 4 m
		Transport	4	15.4	Reisch tipper 12 t

Fendt 724 Gen6	174	Cultivating	1	3.9	Horsch Terrano FX 3 m
		Ploughing	5	10	Lemken Europal 8 with 5 ploughshares
		Silage transport	2	12.5	Fliegl Agroliner 16 t

### 3.1 *{Tractor model}*.csv and *{Field_id}.csv*

Whereas the *{Tractor model}*.csv contains all recorded data from this specific tractor, the *{Field_id}.csv* contains a single field, which is worked with one specific implement. The single fields are identified and separated from the data by the methodology in section 0.

For this study, the data includes information from the J1939 CAN bus and the ISOBUS filtered by the Suspect Parameter Numbers (SPN) and Parameter Group Numbers (PGN) from the J1939 and ISO 11783 standard, as shown in Table 2. Due to the different tractors, their configuration, and age not every parameter might be available for each tractor. The signals include engine-relevant parameters to obtain the load, geospatial data about position and speed, and information about the control of the implementation as 3-point hitch and PTO.

**Table 2:** Description of information contained in the *{Tractor model}.csv* and *{Field_id}.csv.*

Signal	Unit	PGN / SPN	Description
Time	-s		The timestep is 0.1 s. The time is falsified for reasons of anonymity and starts counting from 0, but reflects when work was done at different days.
Index	-		Indication of the lines.
AccelPedalPos1	%	91	The ratio of actual position of the analog engine speed/torque request input device (such as an accelerator pedal or throttle lever) to the maximum position of the input device.
ActualEngPercentTorque	%	513	Calculated output torque of the engine. The engine percent torque value will not be less than zero and it includes the torque developed in the cylinders required to overcome friction.
Ambient Air Temperature	°C	171	Temperature of air surrounding vehicle.
Altitude	m	580	Altitude of the vehicle referenced to sea level at standard atmospheric pressure and temperature.
CourseOverGround	rad		The direction of the path over ground actually followed by a vessel.
EngCoolantTemp	°C	110	Temperature of the engine coolant in the cooling system of a vehicle's engine.
EngFuelRate	L/h	183	Amount of fuel consumed by engine per unit of time.
EngOilPress	kPa	100	Gage pressure of oil in engine lubrication system as provided by oil pump.
EngPercentLoadAtCurrent Speed	%	92	The ratio of actual engine percent torque (indicated) to maximum indicated torque available at the current engine speed, clipped to zero torque during engine braking.
EngReferenceTorque	Nm	544	This parameter is the 100 % reference value for all defined indicated engine torque parameters. It is only defined once and doesn't change if a different engine torque map becomes valid.
Eng_Speed	rpm	190	Actual engine speed which is calculated over a minimum crankshaft angle of 720 degrees divided by the number of cylinders
EstimatedCurvature	1/km	5238 ISOBUS	Machine steering system's estimate of the curvature of the current turn.
FrontAxleSpeed	m/s	904	The average speed of the two front wheels.
Front Draft	N	1878 ISOBUS	Apparent horizontal force applied to the front hitch by an implement. A positive value indicates the force applied to the tractor opposed to its forward direction of travel.
Front Hitch In-work Indication	Bit	1876 ISOBUS	Measured signal indicating that the front hitch is positioned below (in-work) or above (out-of-work) an adjustable switching threshold.
Front Hitch Position	%	1872	Measured position of the front three-point hitch; it is expressed as a percentage of full travel: 0 % indicates the full down position; 100 %, the full up position.
FrontNominalLowerLinkForce	%	1880 ISOBUS	Measurement providing an indication of draft at the lower links of the front three-point hitch.
GroundBasedMachineSpeed	m/s	1859 ISOBUS	Actual ground speed of a machine, measured by a sensor such as that is not susceptible to wheel slip (e.g. radar, GPS, LIDAR, or stationary object tracking).
Latitude	°	584	Latitude position of the vehicle.
Longitude	°	585	Longitude position of the vehicle.
MachineSelectedSpeed	m/s	4305 ISOBUS	This parameter reports the value of one of the currently available machine speeds (wheel-, ground-, or navigational-based), which the machine has determined to best represent the machine's speed.
RearDraft	N	1879 ISOBUS	Apparent horizontal force applied to the rear hitch by an implement. A positive value indicates the force applied to the tractor opposed to its forward direction of travel.
RearHitchInWorkIndication	Bit	1877 ISOBUS	Measured signal indicating that the rear hitch is positioned below (in-work) or above (out-of-work) an adjustable switching threshold.
RearHitchPosition	%	1873 ISOBUS	Measured signal indicating that the rear hitch is positioned below (in-work) or above (out-of-work) an adjustable switching threshold.
RearNominalLowerLinkForce	%	1881 ISOBUS	Measurement providing an indication of draft at the lower links of the rear three-point hitch.
RearPTOOutputShaftSpeed	rpm	8764 ISOBUS	This parameter is used to report the output shaft speed of the rear PTO.
SpeedOverGround	m/s	ISOBUS	Actual ground speed of a machine, measured by a sensor such as that is not susceptible to wheel slip (e.g. radar, GPS, LIDAR, or stationary object tracking).
WheelBasedMachineSpeed	m/s	1862 ISOBUS	The value of the speed of a machine as calculated from the measured wheel or tail-shaft speed.
Work type	String		Type of work which was done during the operation.
Tractor model	String		Tractor model which got recorded.
Implement model	String		Implement specifications that were used during the operation.
Implement width	m		Implement width
Status	String		Indication if the tractor was during on-road or off-road use according to the presented methodology.

### 3.2 Data Quality

**Fig. 1:**
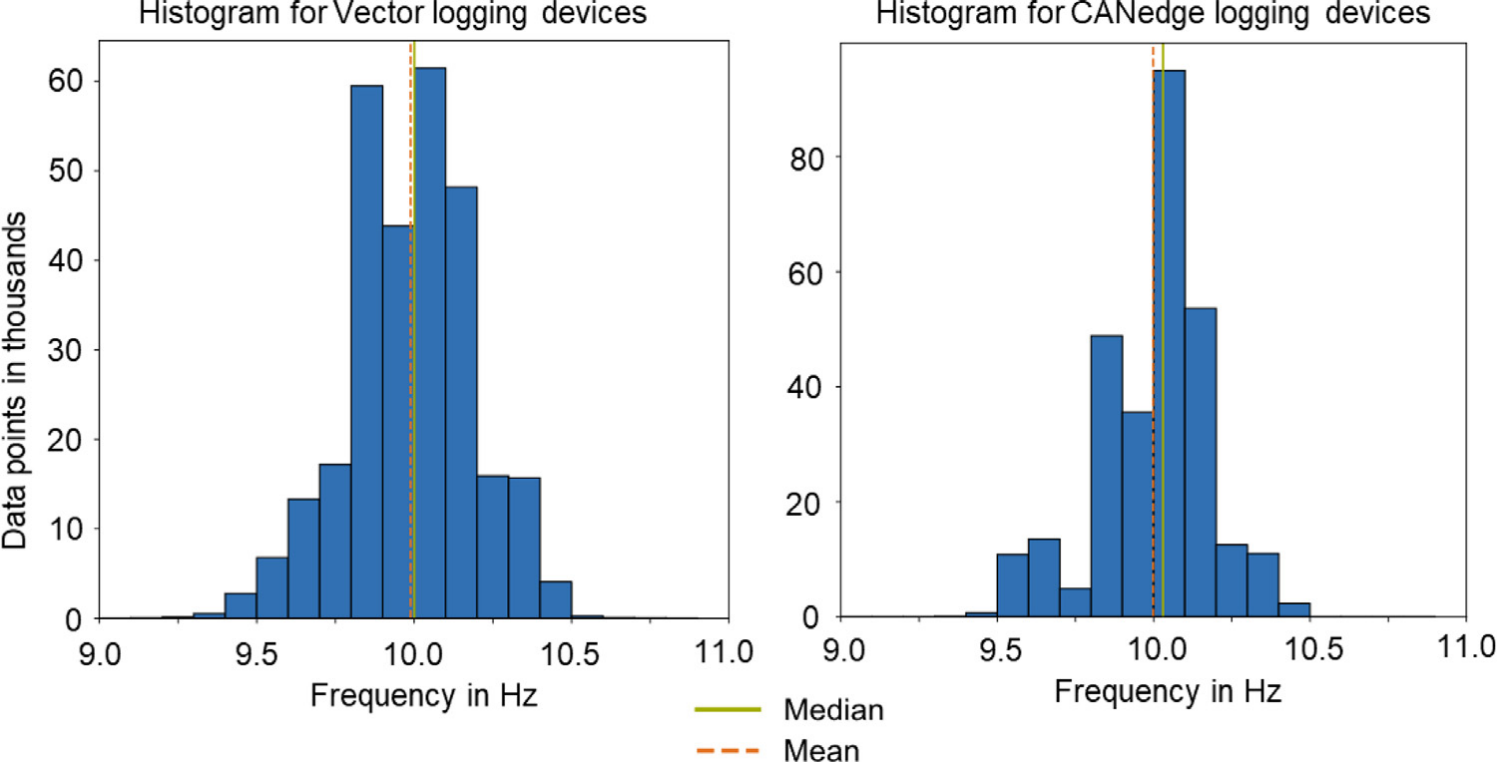
Distribution of raw data logging frequency from the Vectorlogger VN1000 and the CANedge 2 & 3 devices.

The data from both the Vector VN1000 and CANedge 2&3 logging devices is captured with a frequency of 10 Hz. Due to limitations of the data logging devices, the data is not recorded consistently at exactly 10 Hz. Fig. 1 shows the distribution of the frequencies at which the data logging devices recorded the data from a sample of 300 thousand data points. The results show that the logging device from Vector has small deviations in recording frequency, with an average of 9.99 Hz and a median of 10 Hz. The sample of the CANedge device reaches an average frequency of 10 Hz and a median frequency of 10.03 Hz. To ensure consistent timestamps when merging and filtering the data, we resample the data to a uniform frequency of 10 Hz with the Python-integrated method of nearest neighbor for missing values.

### 3.3 Data Statistics

**Fig. 2:**
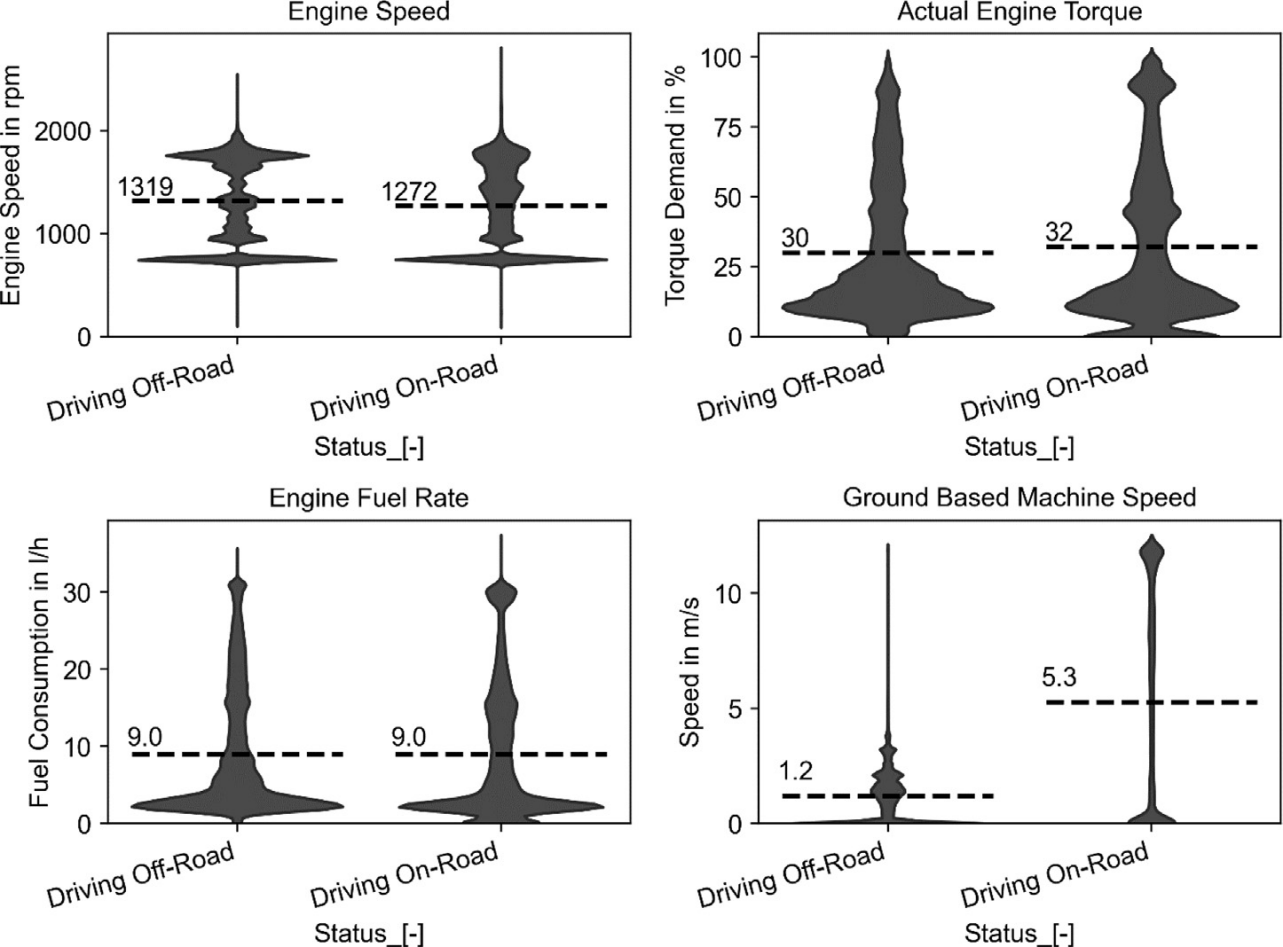
Recorded engine speed, actual motor torque, fuel consumption, and tractor speed distribution for the Fendt 314 during the recorded period split into fieldwork (off-road) and commuting (on-road). The dashed line shows the average.

The published dataset includes a comprehensive number of tasks during the yearly operation of a tractor. To eliminate the effect of the organization and arrangement of the specific recorded farm, we split the data into fieldwork (off-road) and commuting (on-road) operations. The number of commuting operations is individual for every farming operation, but it is significantly important to estimate the efficiency and economics of the operation, as well as the requirements of suitable tractors, e.g., maximum required speed. Hence, driving off-road is defined as any instance where the tractor is not moving on a road, and not idling for more than three minutes. This includes all activities such as working in the field and on the farm, and driving around the farm or within the field. Fig. 2 shows the distribution of engine load-relevant parameters for the Fendt 314 during the recordings. It becomes evident that the fieldwork is exclusively done with speeds below 15 km/h and an average speed of 4.6 km/h. During transport, the tractor either drives at low speeds or close to the maximum speed of 40 km/h, which indicates that road transport is an important design criterion for tractors. The engine speed is concentrated at specific speeds during fieldwork, as PTO implements usually require exactly one engine speed, which emphasizes the need to consider frequent load points at these speeds. The actual engine torque is representative of the operation of this specific tractor, which is mainly used for light tasks with an average load of 30 % during fieldwork. The torque while driving on-road is even slightly higher at 32 % higher but requires the same fuel consumption.

**Fig. 3:**
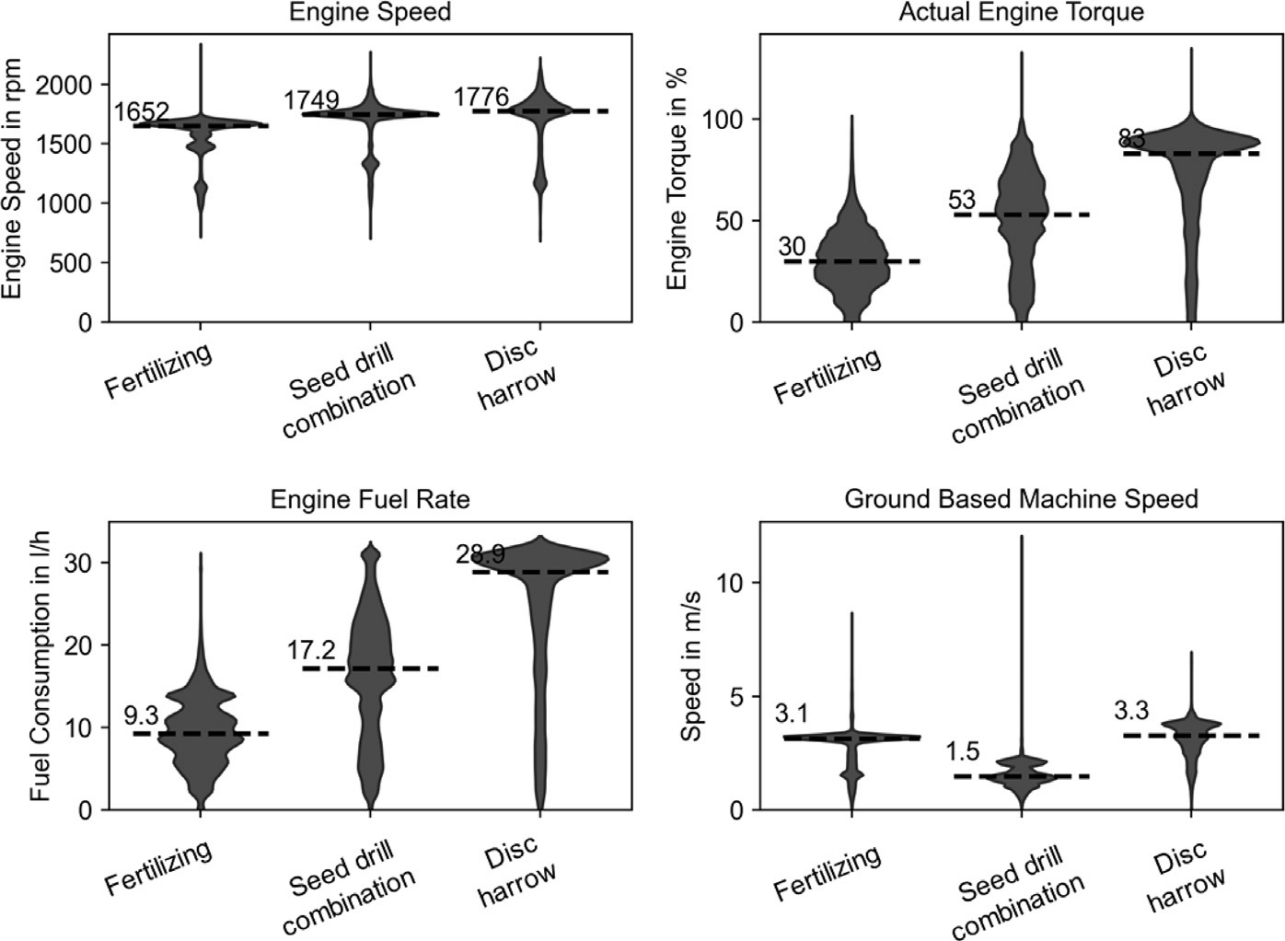
Distribution of engine fuel rate and the ground-based machine speed for the Fendt 314 during the different operations of fertilizing, seed drilling, and disc harrowing.

The recorded engine loads during field operation depend heavily on the used tractor and the attached implements. The presented dataset contains all relevant information to retrieve tractor power information, the used implement, and especially the width of the implement, which are, in most cases, the main determinant for its load requirements. Fig. 3 shows exemplary engine speed and torque with associated fuel rate and ground-based machine speed for the operation of fertilizing, seeding, and discing. While crop care tasks such as fertilizing require low power and low fuel consumption, tillage tasks are very energy intensive, with the required percentage engine torque matching the fuel consumption distribution. The three tasks have a similar required engine speed but differ greatly in torque demand, reflecting the requirement for tractors to deliver a wide distribution of torque. Despite a working width of 15 meters when fertilizing, sowing and disc harrowing are much more energy-demanding at 3 meters implement width.

### 3.4 Level of Detail

**Fig. 4:**
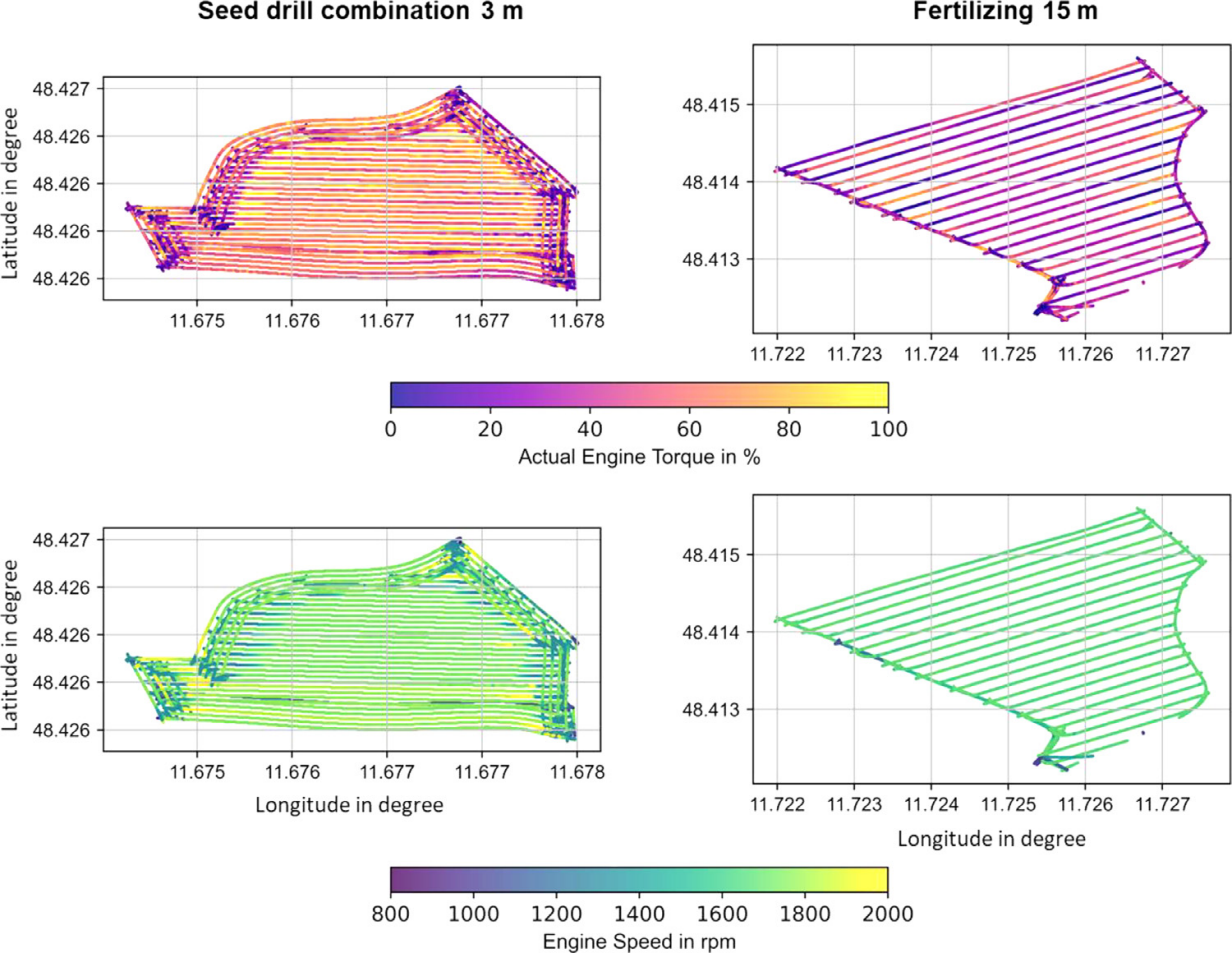
*Actual Engine Percentage Torque* (SPN 513) and *Engine Speed* (SPN 190) for the Fendt 314 during 3 m seed drill combination operation and 15 m fertilizing operation.

Fig. 4 shows two different fields with two different operations of the Fendt 314. The tractor carries out the tasks of seeding with a combination of power harrow and seeder (Table 1) and fertilizing by operating parallel swaths next to each other and turning at the headland. Both implements require to be run by the PTO. In addition to the GNSS position during the operation, the graphs show the necessary load to carry out the tasks of seeding and fertilizing, as shown by the actual engine percentage torque and engine speed signal. The seeding operation requires a median load of 53 % and an engine speed of 1734 rpm; the fertilizing requires a load of 34 % in combination with 1670 rpm on average. Due to the nature of the seed drill combination, which is pulled through the soil by the tractor and simultaneously supplies the power harrow with energy via the PTO shaft, a significantly higher engine torque is required than for the fertilizer spreader, which is also connected to the PTO shaft. The PTO speed is directly linked to the engine speed; the driver, therefore, keeps the engine speed constant during fertilizing to ensure constant fertilizer distribution. A future design of electric drivetrains allows the uncoupling of the direct linkage between the drivetrain and PTO, allowing the operation of each drive at the optimal rotational speed.

**Fig. 5:**
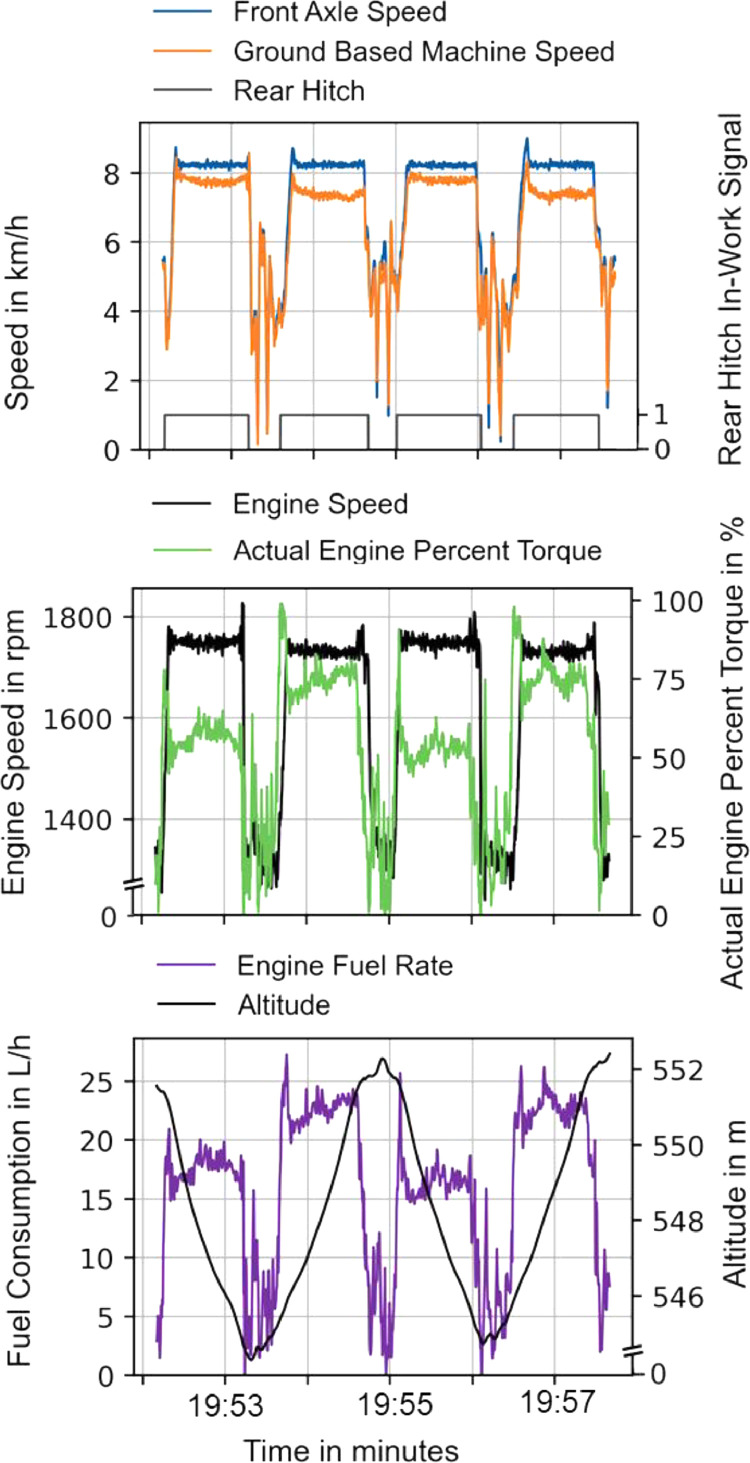
Four swaths of sowing from Fig. 4 on a sloped field: Difference in axle speed and ground-based speed, the necessary engine torque and speed with the according fuel consumption.

Fig. 5 shows a detailed extract of four swaths including the turns from the seeding operation in Fig. 4.

The rear hitch indicator shows the time when the seed drill is in operation. The front axle speed is kept relatively constant, but the actual ground-based speed differs due to slippage. The data in our dataset contains information about the appearing slip due to the recording of the front axle-based speed, which contains slip losses, and the ground-based speed, which is recorded with RTK GNSS.

The engine speed–in black–is kept relatively constant during operation to keep the PTO speed constant. However, the actual load fluctuates, as can be seen by the demanded actual engine torque which correlates with the actual fuel consumption. The direct coupling of the engine and PTO speed in diesel tractors means that the driveline cannot react to the load, as would be possible with electric drives.

Between the swaths, a constant offset appears: The ground-based speed, the engine torque, and the fuel consumption varies from swath to swath with an offset due to the slope of the field and the tractor climbing and descending during operation, demonstrating the consistency of the recorded data.

## 4 Experimental Design, Materials and Methods

**Fig. 6:**
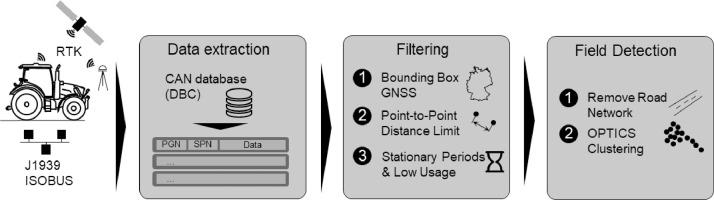
Methodology to derive the data set.

During the agricultural season of 2024, we installed data loggers on five tractors in the power range of 77–240 kW of the brand Fendt (AGCO GmbH, Marktoberdorf, Germany) which were operated on the university farms of the Technical University of Munich (TUM) to cultivate cereals (corn, wheat, soybeans), grassland, and silage production. Fig. 6 guides us through the process of creating the data set.

The bus loggers were connected to the Society of Automotive Engineers (SAE) standard J1939 and the ISO 11783 standard (ISOBUS) with data loggers from the type Vector VN1000 (Vector GmbH, Stuttgart, Germany) and CANedge 2 & 3 (CSS Electronics, Aabyhoej, Denmark). The data loggers recorded the bus data constantly with a frequency of 10 Hz, either when the tractor's 12 V power supply is turned on, or the engine speed is recognized to be above 900 rpm. The GNSS location of the tractor is recorded from the ISOBUS, which includes the corrected Latitude (SPN 584) and Longitude (SPN 585) signal with real-time kinematic (RTK) corrections with an accuracy of ± 2 cm. The Fendt 820 falls back on the internal GNSS signal from the CANedge 2 logger without any correction due to a missing GNSS system on the tractor.

## 5 Data Extraction and Filtering

The data filtering differed depending on the logger type. For the CANedge logging devices, the data was extracted in the binary raw measurement format (MF4) and decoded within Python with the CAN database files (DBC) for J1939 and ISOBUS with an open-source package^2^ (see data extraction in Fig. 6). For the Vector logging devices, the data was retrieved in the Common Log Format (CLF) format and directly decoded in the proprietary Vectorlogger Suite program with the DBC databases. The data cleaning and filtering processes for both data logging types converged into a unified pipeline in Python. The dataset is filtered only for signals relevant to this dataset, which we defined in Table 1.

Still, the process contains data points with corrupted GNSS data due to shading and limited satellite or RTK availability. To remove corrupted data points, we approach the following two methods: (1) A bounding box of Germany defines the maximum and minimum coordinate values that are allowed. Values outside the bounding box are corrupted and removed. (2) In addition, we introduce a distance threshold filter between consecutive points. The distance d between the coordinates of two consecutive data points is determined using the haversine formula and must be less than 10 m, which corresponds to the maximum speed of the tractor including a buffer. Otherwise, the data points are filtered out:

$$d=2R_{E}\cdot \arcsin\left(\sqrt{\sin^{2}\left(\frac{\Delta \phi }{2}\right)+\cos\left(\phi_{1}\right)\cdot \cos\left(\phi_{2}\right)\cdot \sin^{2}\left(\frac{\Delta \lambda }{2}\right)}\right)\;<\;10\;m$$

with the earth radius $R_{E}$, Δϕ and Δλ being the differences in latitude and longitude and the latitude and longitude coordinate $\phi_{1}$ and $\phi_{2}$.

After we removed corrupted data points, we limited the data to the operation of the tractor. Stationary periods, where the tractor's velocity and engine speed are both zero for more than 30 min are removed. In addition, days with an operating time of less than 30 min are deleted because, after consultation with the operators, this indicates alternative use of the tractor, e.g. for trips to workshops or for organizational tasks. The last step includes merging completely identical data points. With a moving box of one second, identical data points are merged. If more than four consecutive data points reflect default values according to Table 2, they are set to *nan*. The data points are then published in the *{Tractor model}.csv.*

## 6 Field Detection

After we extracted the data and removed corrupted data, the last step included the field detection process (Field Detection in Fig. 6). Our data set enables the separation between fieldwork (off-road) and commuter (on-road) operations and, thus, the investigation of the tractor load caused by various implements exclusively during fieldwork. Therefore, we compare the coordinates of the recorded data points with the coordinates from the German road network from Open Street Map^3^. We removed the coinciding road network with a buffer of 10 m to account for GNSS inaccuracies.

**Fig. 7:**
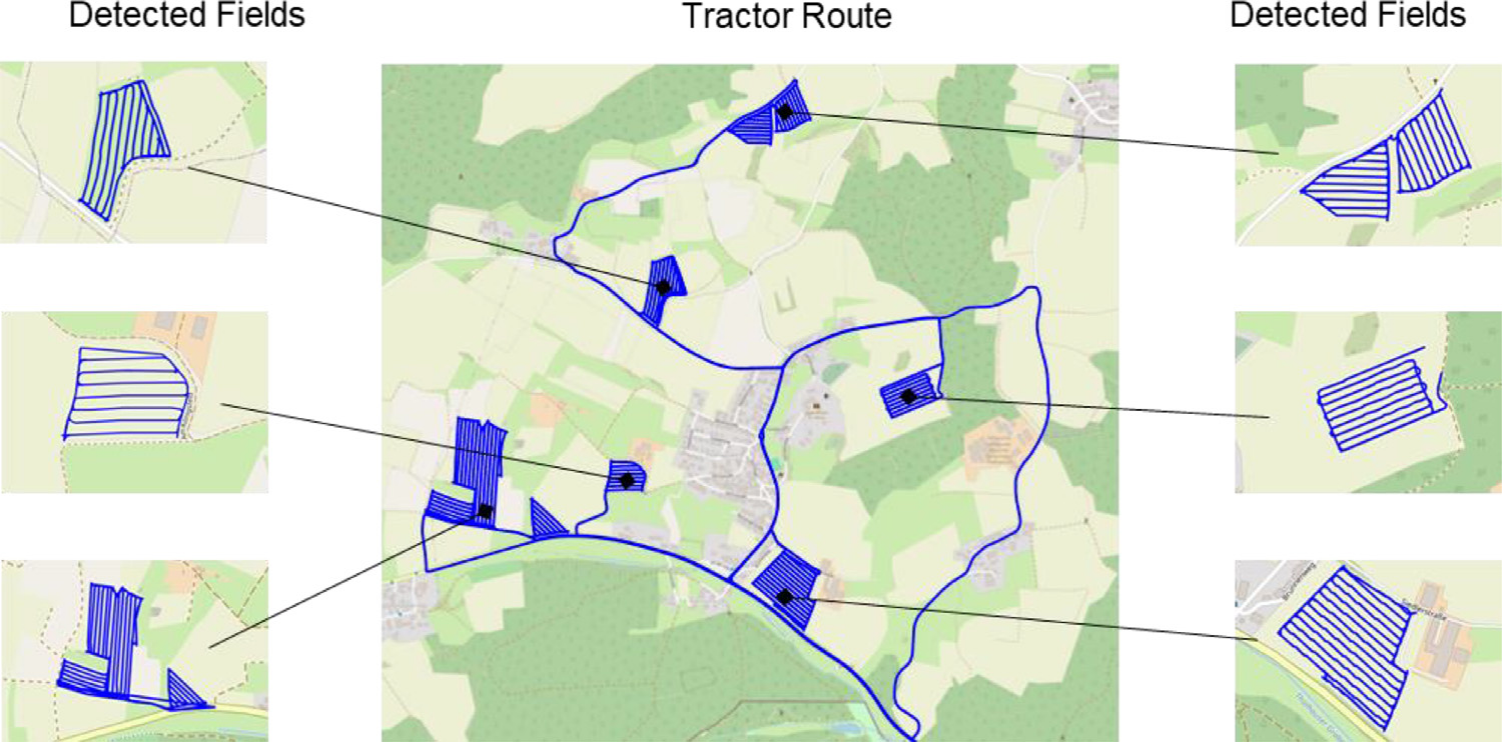
Exemplary tractor route of the Fendt 314 during a day-long seed drill operation with the automatically detected fields.

The data containing multiple fields must be split per field, as can be seen in Fig. 7. Unlike other authors as Mattetti et al. [16], who need field boundaries to detect the fields, we further developed the approach of Chen et al. [17] to automatically detect the fields:

We take advantage of the characteristic of fieldwork that generates a high density of data points in the field segment during operation, combined with the occurrence of low operating speeds. Therefore, we use the density-based clustering method Ordering Points To Identify the Clustering Structure (OPTICS). The clustering method is based on the nearest neighbour method in determining whether a data point is determined as part of a cluster. For our approach, the algorithm showed the best results with a maximum distance between two points of 30 m. The results were validated by assuring a detected field contains at least 2000 data points corresponding to more than 200 s and an average speed within the field between 0.3 and 3.5 m/s, which are reasonable to the operation according to the operators. The data points of the field segments are then published in the *{Field_id}.csv.*

## Limitations

The recorded operations reflect agricultural activities at the university farm of the Technical University of Munich (TUM). The general mission profile and the division of time between commuter and off-road activities are specifically determined by this particular operation.

## Ethics Statement

The authors have read and followed the ethical requirements for publication in Data in Brief and confirm that the current work does not involve human subjects, animal experiments, or any data collected from social media platforms.

## CRediT authorship contribution statement

**Korbinian Götz:** Conceptualization, Methodology, Formal analysis, Investigation, Writing – original draft, Writing – review & editing, Visualization. **Andrew Kusuma:** Software, Data curation, Visualization, Writing – review & editing. **Adrian Dörfler:** Software, Data curation, Visualization, Writing – review & editing. **Markus Lienkamp:** Resources, Writing – review & editing, Supervision.

## Data Availability

(Zenodo)Agricultural Load Cycles: Tractor Mission Profiles From Recorded Gnss and Can Bus Data (Original data).

## Footnotes

1 Nonroad Transient Cycle.

2 CSS-Electronics GitHub. "CSS-Electronics/can_decoder: API module for decoding raw CAN bus data to physical values." Accessed: Dec. 3, 2024. [Online]. Available: https://github.com/CSS-Electronics/can_decoder.

3 OpenStreet Map Foundation (OSMF). "OpenStreetMap." Accessed: Dec. 9, 2024. [Online]. Available: https://www.openstreetmap.org/history.
